# Urban Quality and Biochemical, Hematological, and Nutritional Markers in Older Adults: Cross-Sectional Geospatial Study

**DOI:** 10.2196/74313

**Published:** 2025-10-27

**Authors:** Carlos Mena, Yony Ormazabal, Nacim Molina, Eduardo Fuentes, Juan Carlos Cantillana, Victoria Villalobos, Moises Sandoval, Ivan Palomo, Diego Arauna

**Affiliations:** 1 Longevity Center VITALIS, Faculty of Economics and Business, University of Talca, Avenida Lircay S/N, Talca, Chile, Talca, Chile; 2 Thrombosis Research Center, Department of Clinical Biochemistry and Immunohematology, Faculty of Health Sciences, Interuniversity Center of Healthy Aging (CIES), Longevity Center VITALIS, University of Talca, Avenida Lircay S/N, Talca, 3460000, Chile, +56 712200200; 3 Faculty of Administration and Economics, Universidad Tecnológica Metropolitana, Santiago, Chile; 4 Instituto de Nutrición y Tecnología de los Alimentos (INTA), University of Chile, Santiago, Chile

**Keywords:** aging, frailty, geospatial analysis, urban quality, hematological parameters, nutritional status, biochemical parameters

## Abstract

**Background:**

The urban environment is an important determinant of frailty, primarily through factors such as infrastructure that supports physical activity, availability of social and medical support, and access to nutritious food. Given the increasing aging population, understanding the link between urban quality, frailty, and metabolic health is crucial for effective public health and urban planning interventions.

**Objective:**

This study aims to quantify the impact of distinct urban domains (built-environment characteristics, accessibility to essential services, availability of green and recreational spaces, and neighborhood socioeconomic context) on frailty status, nutritional profile, and hematological or biochemical biomarkers in community-dwelling older adults by integrating geospatial analysis.

**Methods:**

A cohort of 251 older adults (aged older than 65 years) was studied. Frailty was assessed using the Frailty Trait Scale 5, and nutritional status was determined using the Controlling Nutritional Status score. Hematological and biochemical parameters were evaluated in a subset of 70 participants by MINDRAY automatic equipment. A spatial analysis of frailty was conducted by incorporating Geographic Information System layers that mapped the distribution of urban facilities, including fruit and vegetable shops, senior centers, pharmacies, emergency health centers, parks and squares, community centers, and exercise facilities. Statistical analyses included *t* tests, Mann-Whitney *U* test, ANOVA, and correlation analyses.

**Results:**

The prevalence of frailty was 17.5%. Frail individuals exhibited significantly higher BMI (mean 31.5, SD  4.4 vs mean 28.5,  SD 4.5 kg/m²; *P*=.0001). When comparing the upper (Q4) and lower (Q1) quartiles of urban quality, Q4 participants had higher Frailty Trait Scale 5 scores (mean 15.2,  SD 7.4 vs mean 11.8, SD  6.4; *P*=.0334) and lower handgrip strength (mean 19.1, SD 4.4 vs mean 22.8, SD 7.3 kg; *P*=.006). Frail individuals resided significantly closer to emergency health centers (*P*=.0010), family health centers (*P*=.0412), and exercise facilities (*P*=.0322). In addition, bilirubin (Spearman ρ=0.33; *P*=.0049), serum iron (Spearman ρ=0.27; *P*=.0272), transferrin saturation (Spearman ρ=0.24; *P*=.0386), red blood cell count (Spearman ρ=0.26; *P*=.0303), and red blood cell distribution width (Spearman ρ=0.23; *P*=.0462) were positively correlated with urban quality. Frail participants also had higher Controlling Nutritional Status scores (*P*=.0323), which were positively correlated with urban quality (Spearman ρ=0.25; *P*=.0359).

**Conclusions:**

Urban quality was significantly associated with hematological parameters, nutritional status, and frailty. Frail individuals in areas with better urban quality exhibit lower handgrip strength, higher frailty scores, and greater proximity to emergency rooms, community health centers, and exercise facilities. This spatial distribution may reflect higher accessibility to health care and recreational resources among frail participants. Urban planning and public health strategies should focus on creating age-friendly environments to prevent frailty and improve health outcomes.

## Introduction

Global demographic changes have indicated an accelerated increase in the aging population, along with a rise in the prevalence of older adults [1]. This demographic transition has prompted the analysis of various key factors associated with the aging process, with frailty being one of the primary foci in geriatric medicine [2]. This multisystemic syndrome is characterized by a decline in physiological reserves and intrinsic capacity, which leads to an increased vulnerability to stressors and a higher risk of adverse outcomes such as falls, hospitalization, disability, and mortality [3,4]. The global prevalence of frailty varies between 12% and 24%, depending on the diagnostic tool used, and it is one of the key factors to prevent and reverse according to the World Health Organization recommendations [5]. The increase in the frailty prevalence among older adults poses significant challenges for health care systems, highlighting the need for comprehensive strategies to address its underlying determinants [2,3,6]. Recent studies have highlighted the significant role of the urban environment in frailty, influencing both its development and improvement, independent of individual factors [7]. The built environment, characterized by access to green spaces, health services, community resources, places for physical activity, diet, and social interactions, plays a key role in frailty [7-9]. This is reflected in various studies indicating that older adults living in areas with lower land-use diversity and more social incivilities may be at risk of developing mobility limitations. In contrast, those residing in urban areas with limited resources and higher pollution levels tend to exhibit higher mortality rates associated with frailty [10,11]. In this context, frailty and mobility limitations have been associated with the neighborhood physical characteristics, including land-use mix diversity, land-use mix access, street connectivity, walking infrastructure, esthetics, crime safety, and social incivilities [11,12]. Due to the relevance of frailty in older adults, the World Health Organization has recognized the need to create age-friendly environments to enhance healthy aging and reduce the risk of frailty [5]. In Latin America, urban inequalities are particularly pronounced, with limited access to critical services and stark disparities in urban quality [13]. Chile exemplifies this context, characterized by rapid urbanization, a pronounced rural-urban divide, and significant socioeconomic heterogeneity. While previous studies have linked environmental factors to frailty in high-income countries [7-9], evidence from Latin America remains limited, particularly concerning the interplay between urban quality, hematological or biochemical markers, and nutritional status in aging populations. Notably, no Chilean study to date has integrated geospatial analysis with clinical biomarkers to examine how specific urban features (eg, proximity to emergency centers or green spaces) correlate with frailty phenotypes. This gap is critical, as Chile’s unique urban-rural dynamics and aging demographics may reveal patterns not observed in more developed settings [14]. In this study, we address this gap by combining Geographic Information System (GIS)–based urban metrics with multidimensional frailty assessments (Frailty Trait Scale 5 [FTS-5]), biochemical or hematological profiling, and nutritional status (Controlling Nutritional Status [CONUT] score) in a Chilean cohort, offering novel insights for regional public health strategies. By integrating GIS data with health and sociodemographic variables, we aim to analyze possible spatial clusters of frailty, assess the impact of urban quality, and highlight the potential of urban planning and policy interventions in mitigating frailty risks [8,9,15]. In addition to environmental factors, nutritional and hematological status is a key component associated with frailty. Urban environmental conditions have also been implicated in modulating blood-based health indicators [16,17]. Hematological abnormalities, such as variations in red blood cell (RBC) count and iron status, are linked to frailty, potentially through their effects on muscle function, oxygen transport, and fatigue. Moreover, several biochemical markers reflecting hepatic and renal function have been associated with frailty in older adults [18]. Understanding how urban quality influences these clinical parameters may provide novel perspectives for developing targeted frailty prevention strategies [17]. With this background, the aim of this study was to quantify the impact of distinct urban domains (built-environment characteristics, accessibility to essential services, availability of green and recreational spaces, and neighborhood socioeconomic context) on frailty status, nutritional profile, and hematological or biochemical biomarkers in community-dwelling older adults by integrating geospatial analysis.

## Methods

### Participants and Study Design

A representative sample of 251 older adults (aged 65 years and older; both men and women) from the older adult population of Talca, Chile (Maule Region), registered with Family Health Centers and community organizations, participated in this cross-sectional study. The inclusion criterion was adults aged 65 years and older. Participants with self-reported or medically documented cancer, Parkinson disease, or vascular accidents were excluded, as well as older individuals unable to walk or speak, and those currently on statin therapy [19]. The sample size calculation was based on an estimated frailty prevalence of 24.6% among older adults, with a 95% confidence level, 80% statistical power, and an anticipated 20% attrition rate. The gender distribution of the sample reflected that of the population aged 65 years and older, according to data from the National Socioeconomic Characterization Survey.

### Frailty Diagnosis

Frailty was measured using the FTS-5, which assesses frailty across 5 key domains: nutrition, physical activity, nervous system, strength, and gait speed. Nutrition was evaluated using BMI [20]. Physical activity was assessed with the Physical Activity Scale for Elderly. Nervous system function was evaluated using the Progressive Romberg Test. Strength was determined by handgrip strength in the dominant hand. Gait speed was measured by time taken to complete a 3-meter walk at a usual pace. Each domain is scored on a scale from 0 to 10 points. Frailty was determined by summing the scores across all 5 domains, resulting in a total range of 0-50 points. A score above 25 was considered indicative of frailty. The FTS-5 Scale has demonstrated high diagnostic accuracy (AUC: 0.81‐0.85 for frailty incidence) in urban-dwelling older adults, with strong psychometric properties relative to Fried’s phenotype (sensitivity 0.608; specificity 0.823) and the FRAIL Scale (sensitivity 0.642, specificity 0.814). Unlike Fried’s criteria, which rely solely on physical characteristics, the FTS-5 incorporates neurophysical and functional dimensions, enhancing its sensitivity in heterogeneous urban populations.

### Nutritional Status

Nutritional status was assessed using the CONUT score, a validated tool based on 3 biochemical parameters: serum albumin, total lymphocyte count, and total cholesterol levels [21]. Each parameter is assigned a score based on predefined thresholds, and the sum of these scores provides the overall CONUT score. Higher scores indicate a greater degree of malnutrition, with classifications ranging from normal (0‐1) to severe malnutrition (≥9).

### Hematological and Biochemical Profile

Seventy plasma and serum samples were randomly obtained from cohort participants. Blood samples were collected using ethylenediaminetetraacetic acid (4.5 mL) and without anticoagulant (5 mL) by phlebotomy. The ethylenediaminetetraacetic acid blood sample was used to determine hematological profile (BC-3600 MINDRAY) using automated equipment. The blood sample without anticoagulant was centrifuged at 240 g for 15 minutes to obtain serum, which was used to determine the biochemical profile (BC-300 MINDRAY) using automated equipment. The sample size was calculated using the GRANMO calculator with a 95% confidence level, 80% statistical power, and an expected loss rate of 20%.

### Geospatial Database

**Figure 1.**
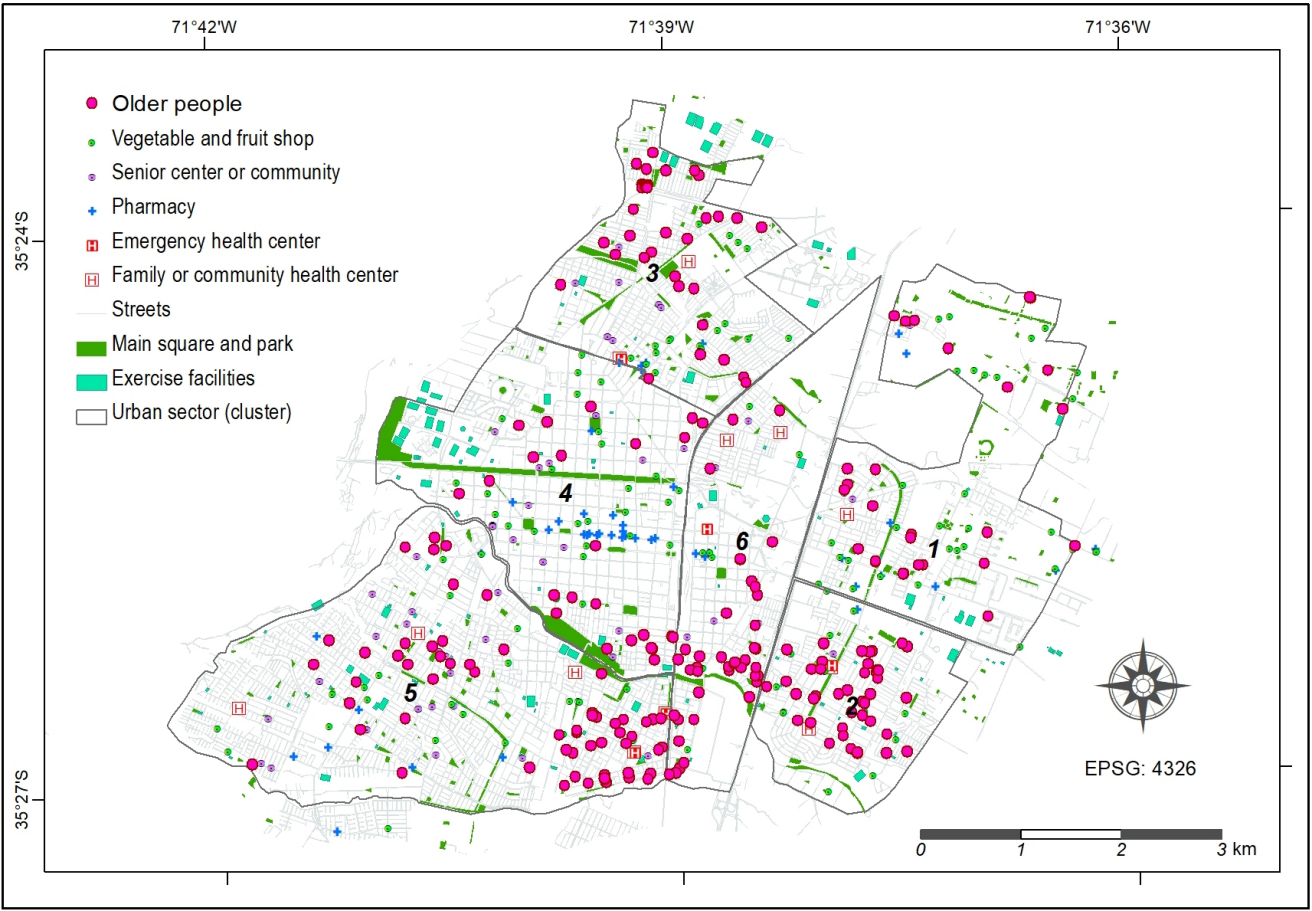
Locations of each older adult in the study within the city of Talca, alongside the available urban physical facilities.

A georeferenced database was created using GIS technology to represent the physical environment of the study area and the locations of the older adults participating in the study (Figure 1). Each participant was geographically located as a point object based on the residence data provided during the medical evaluation. All data were organized into a point feature layer, accompanied by a corresponding thematic table. The database included relevant factors related to urban physical infrastructure within the city, such as fruit and vegetable shops, senior centers or communities, pharmacies, emergency health centers, main squares and parks, family or community health centers, and exercise facilities. Each component was represented as a GIS layer, either in point or polygon form, at the neighborhood level, using geographical information from OSM (OpenStreetMap), Google Maps, and IDE Chile (Geospatial Infrastructure of Chile).

The city of Talca (Maule Region, Chile) was further divided into 6 main clusters based on sociodemographic characteristics: cluster 1 represents the northeastern sector with a high socioeconomic status (SES), cluster 2 covers the southeastern sector with a low SES, cluster 3 includes the northern sector with a lower-middle socioeconomic class, cluster 4 corresponds to the historic center, cluster 5 encompasses the southern sector with a medium-high socioeconomic level, and cluster 6 refers to the industrial center area. These clusters were defined using census data and municipal planning criteria, considering variables such as household income, education level, housing density, and land use. Classification was based on expert-informed manual grouping rather than statistical clustering algorithms.

### Urban Quality Level

Each GIS layer was analyzed using the Euclidean distance method to assess the proximity of every location within the city to various urban facilities. The resulting distance layers were then classified into 3 zones: close, medium, and distant. Each zone was assigned a score on a scale from 1 to 3, with the closest proximity receiving a score of 3 and the most distant areas a score of 1. The distance ranges and corresponding scores for each urban facility were defined based on the local context, as shown in Table 1. These parameters had been previously applied in the same city [14] and are considered representative of 3 distinct levels of access efforts. The general criterion was that closer proximity to a facility indicated higher urban quality. A raster calculator was used to aggregate all layers into a summary index, with higher numerical values reflecting better urban quality in the corresponding area. The facilities included in this index were vegetable and fruit shops, senior centers or communities, pharmacies, emergency health centers, main squares and parks, family or community health centers, and exercise facilities, as detailed in Table 1. The summary index was computed as a continuous raster surface (50-m resolution) covering the entire urban area. Values were extracted at each participant’s georeferenced location using a spatial join. While the index was later analyzed in relation to spatial clusters, its computation was conducted across the full urban extent rather than per cluster. All data management, processing, and analysis were performed using ArcGIS software (version 10; ESRI).

**Table 1.** Distance ranges and corresponding values assigned to each urban physical facility.

Facility	Distance ranges, m		
Vegetables and fruits shops	<300	300‐600	>600
Senior centers or communities	<500	500‐1000	>1000
Pharmacies	<500	500‐1000	>1000
Emergency health centers	<1000	1000‐2000	>2000
Main squares and parks	<200	200‐400	>400
Family or community health centers	<700	700‐1400	>1400
Exercise facilities (stadiums and green areas with urban assets designed for physical activity)	<400	400‐600	>600
	Values		
	3	2	1

### Statistical Analysis

Statistical analyses were conducted using GraphPad Prism 9. Continuous variables were expressed as mean (SD) or median with 95% CI. Categorical variables were expressed as percentages with 95% CI. Group differences were assessed using the Chi-square test with Yate’s correction for proportions. For differences in means or medians, the *t* test, Mann-Whitney *U* test, or ANOVA was applied, depending on the context. Correlation analysis was performed using Spearman correlation coefficient (ρ). Statistical significance was considered at *P* values of <.0500.

### Ethical Considerations

The institutional review board approval for this study was obtained from the Comité de Ética Científica of Universidad de Talca (reference number 06-2021). All procedures adhered to the ethical standards of the Comité de Ética Científica and the World Medical Association’s Declaration of Helsinki. All participants provided written informed consent. All data were deidentified; stored on encrypted, password-protected institutional servers with access restricted to authorized personnel; and reported only in aggregate. Geospatial data were coded to administrative units to minimize reidentification. Participants participated voluntarily and received no compensation.

## Results

### Sociodemographic Characteristics and Spatial Cluster Distribution by Frailty Status

Table 2 shows the sociodemographic characteristics of the study cohort, as well as the geospatial distribution of nonfrail and frail individuals across the spatial clusters (1-6). The cohort consists of 17.5% (44/251) frail older adults and 82.5% (207/251) nonfrail older adults. No significant differences were observed in the prevalence of women between groups (*P*=.642). The frail group had a higher mean age (mean 75.6, SD 7.4 years) than the nonfrail group (mean 73.8, SD 5.2 years); however, this difference was not statistically significant (*P*=.0583). Conversely, the frail group exhibited a significantly higher mean BMI (mean 31.5, SD 4.4 kg/m²) than the nonfrail group (mean 28.5, SD 4.5 kg/m²) (*P*=.0001). Regarding geospatial distribution, no significant differences were observed between the groups.

**Table 2.** Sociodemographic description and geospatial distribution of the studied sample of older people according to frailty status.

	Nonfrail (n=207)	Frail (n=44)	*P* value
Variables			
Female % (95% CI)	73.9 (67.5‐79.4)	77.2 (63.0‐87.1)	.6424
Age (years), mean (SD)	73.8 (5.2)	75.6 (7.4)	.0583
BMI, mean (SD)	28.5 (4.5)	31.5 (4.4)	.0001
Spatial cluster			
Cluster 1 (95% CI)	12.6 (8.7‐17.7)	6.8 (2.3‐18.2)	.2792
Cluster 2 (95% CI)	17.4 (12.8‐23.1)	18.2 (9.5‐31.9)	.5918
Cluster 3 (95% CI)	17.9 (13.2‐23.6)	22.7 (12.8‐36.9)	.4536
Cluster 4 (95% CI)	11.6 (7.9‐16.7)	6.8 (2.3‐18.2)	.3531
Cluster 5 (95% CI)	23.1 (17.9‐29.4)	27.3 (16.3‐41.8)	.5640
Cluster 6 (95% CI)	17.4 (12.8‐23.1)	18.2 (9.5‐31.9)	.5918

### Frailty Characterization According to Quartile Distribution of Summary Index

**Figure 2.**
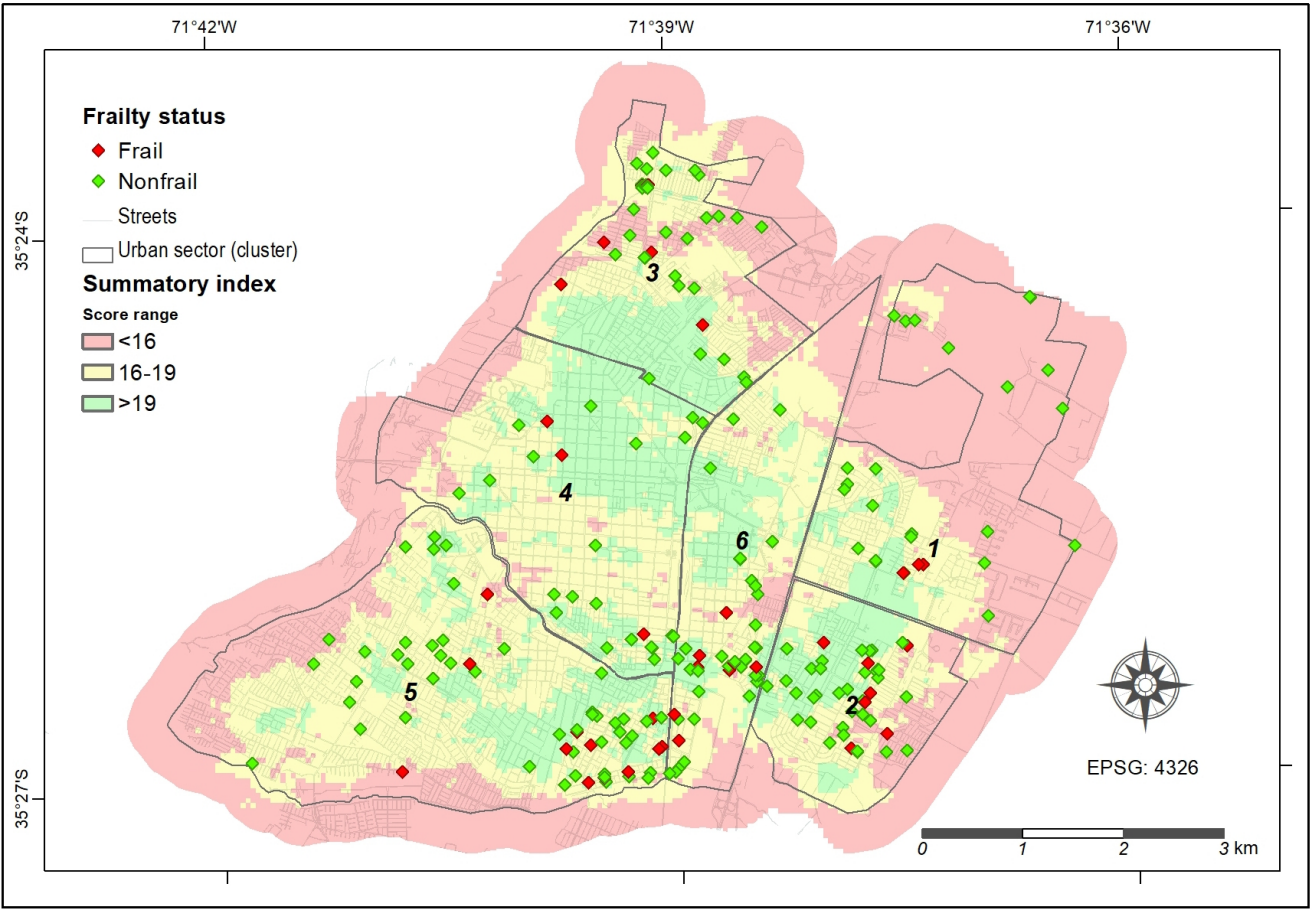
Frailty status of older adults across the summary index within the geospatial clusters defined for the city of Talca.

Figure 2 illustrates the different levels of urban quality based on a cumulative assessment (summatory index) of physical environmental factors considered in this study. These factors primarily encompass essential urban services and infrastructure relevant to the older adult cohort under investigation. Urban quality levels are closely linked to the accessibility of various facilities from each location within the city. In addition, Figure 2 displays the spatial distribution of individual participants, with each one identified by frailty status.

Table 3 shows mean values for frailty prevalence, frailty score, handgrip strength, gait speed, and BMI, grouped by quartiles of the urban quality summary index. While spatial clusters represent areas with homogeneous socioeconomic and urban characteristics (eg, cluster 1 corresponds to a high SES area in the northeast), the quartiles (Q1-Q4) classify areas based solely on their objective accessibility to services, regardless of SES. As shown in Figure 2, for instance, cluster 5 (classified as medium-high SES) includes both Q1 (poorest access) and Q4 (best access) zones, highlighting that even socioeconomically advantaged areas may contain localized disparities in service provision. As previously described, lower values on the summary index indicate poorer urban quality, while higher values reflect better urban quality. Q1 represents the quartile with the lowest index values (poorest urban quality), and Q4 represents the highest values (best urban quality). No statistically significant differences were observed across quartiles for the analyzed variables concerning frailty prevalence (*P*>.0500). However, when comparing Q4 with Q1, participants in Q4 exhibited a significantly higher FTS-5 score (mean 15.2, SD 7.4 vs mean 11.8, SD 6.4; *P*=.0334) and a significantly lower handgrip strength (mean 19.1, SD 4.4 vs mean 22.8, SD 7.3; *P*=.0059). No significant differences were found between Q4 and Q1 for frailty prevalence, gait speed, or BMI (*P*>.0500).

**Table 3.** Frailty prevalence, handgrip strength, gait speed, and BMI according to quartile distribution of summary index.

Frail status	Q1 (12-17)	Q2 (>17‐19)	Q3 (>19‐20)	Q4 (>20‐22)	*P* value
Frail % (95% CI)	15.3 (8.7‐25.3)	17.8 (11.6‐26.4)	17.3 (9.4‐29.7)	23.1 (11.0‐42.0)	.8462
FTS-5 Scale score, mean (SD)	11.8 (6.4)	13.6 (7.9)	13.7 (8.3)	15.2 (7.4)^a^	.2092
Handgrip strength (kg), mean (SD)	22.8 (7.3)	21.3 (7.3)	22.4 (7.2)	19.1 (4.4)^a^	.2620
Gait speed (m/s), mean (SD)	1.0 (0.3)	0.9 (0.2)	0.9 (0.2)	0.9 (0.2)	.6593
BMI (kg/m^2^), mean (SD)	29.3 (4.3)	29.4 (4.1)	29.1 (4.4)	28.5 (4.2)	.7723

a *P* value<.0500 respect to Q1.

b FTS-5: Frailty Trait Scale 5.

### Relationship Between Frailty and Distances to Urban Facilities and Urban Quality

**Figure 3.**
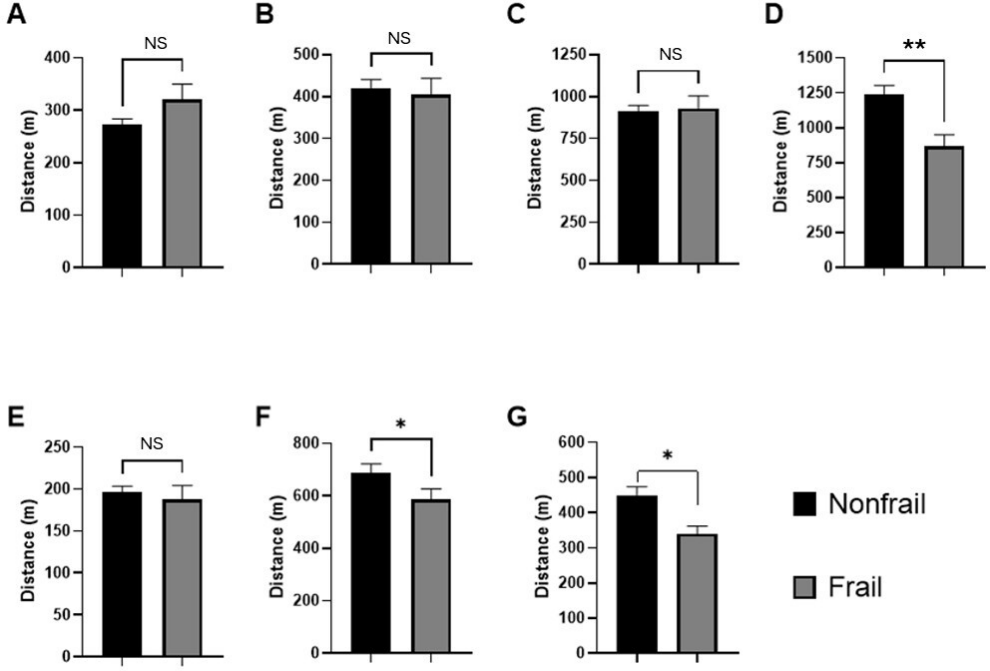
Comparison of mean distance for different frailty status diagnosed by Frailty Trait Scale 5 Scale with respect to relevant urban facilities: (A) vegetables and fruits shops, (B) senior centers or communities, (C) pharmacies, (D) emergency health centers, (E) main squares and parks, (F) family or community health centers, and (G) exercise facilities. The data presented are mean (SEM). Statistical analysis was performed using the *t* test or Mann-Whitney *U* test, as appropriate. *P<.0500. **P<.0001. NS: not significant.

Figure 3 shows a comparison of average distance to urban facilities by frailty status, as assessed using the FTS-5 clinical tool. No significant differences were observed in the distance between frail and nonfrail groups for key urban areas such as vegetable and fruit shops (Figure 3A), senior centers or communities (Figure 3B), pharmacies (Figure 3C), and main squares and parks (Figure 3E) (*P*>.0500). However, Figure 3A suggests a trend where frail individuals tend to live further from vegetable and fruit shops. In contrast, Figure 3D shows that the frail group is significantly closer to emergency health centers (*P*=.0010). Similarly, the frail group was significantly closer to family or community health centers (Figure 3F) (*P*=.0412) and stadiums and exercise facilities (Figure 3G) (*P*=.0322).

**Figure 4.**
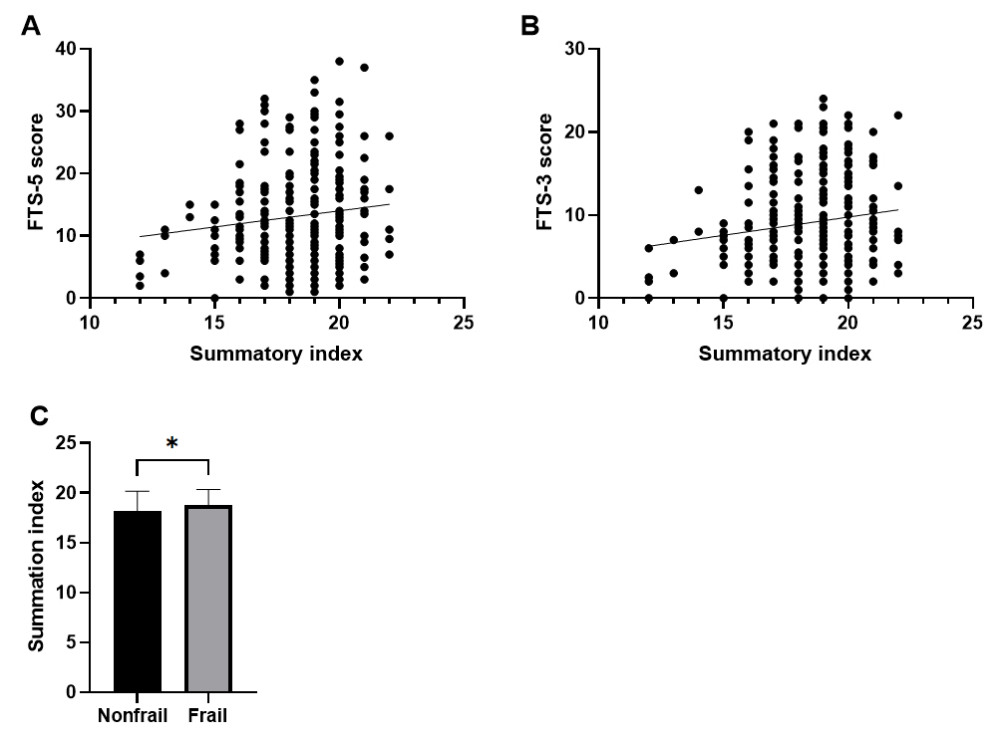
Linear regression between frailty scores and summary index, and comparison of the summary index by frailty status. (A) Linear regression between summary index and FTS-5 score, (B) linear regression between summary index and FTS-3 score, and (C) comparison of mean summary index by frailty status diagnosed by FTS-5. The data presented are mean (SEM). Statistical analysis was performed using the *t* test or Mann-Whitney *U* test, as appropriate. *P<.0500. FTS: Frailty Trait Scale.

Figure 4 shows the correlation analysis between frailty scores and the summary index and compares this parameter by frailty status. In Figure 4A, the linear regression between the summary index and the FTS-5 score shows a positive correlation, where an increase in the summary index corresponds to a higher FTS-5 score. This correlation is statistically significant, with a Pearson *r* of 0.1319 (*P*=.0368). A similar trend is observed in Figure 4B, which illustrates the correlation between the summary index and the FTS-3 score (a shortened version of FTS-5), showing a Pearson *r* of 0.1562 (*P*=.0132). Figure 4C compares the summary index by frailty status, demonstrating a modest but significant increase in the frail group (*P*=.0310).

### Relationship Between Urban Quality and Biochemical and Hematological Parameters

**Figure 5.**
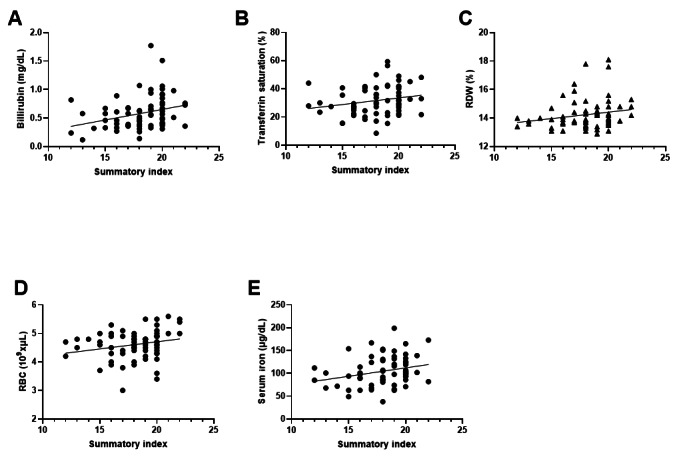
Linear regression analysis between summary index and hematological and biochemical parameters. (A) Bilirubin, (B) transferrin saturation, (C) RDW, (D) RBC, and (E) serum iron. Statistical analysis was performed using linear regression and Spearman correlation analysis. RBC: red blood cell count; RDW: red blood cell distribution width.

Table 4 shows the results of the correlation analysis between the summary index and the biochemical and hematological parameters of the older adult participants. At the biochemical level, only bilirubin levels showed a significant correlation with the summary index (Spearman ρ=0.3325) (*P*=.0049). Figure 5A displays the linear regression for this parameter, illustrating a direct relationship where higher summary index values are associated with increased bilirubin levels in older adults. At the hematological level, several parameters showed a significant correlation with the summary index. Serum iron exhibited a positive correlation (Pearson *r*=0.2720; *P*=.0272), as illustrated in Figure 5E, where higher summary index values correspond to increased serum iron levels. Similarly, transferrin saturation (Spearman ρ=0.2478; *P*=.0386), RBC count (Pearson *r*=0.2629; *P*=.0303), and red blood cell distribution width (RDW) (Pearson *r*=0.2391; *P*=.0462) also displayed positive correlations with the summary index. The linear regression graphs in Figure 5B-E, corresponding to each parameter, respectively, further illustrate this direct relationship, where an increase in the summary index was associated with higher values of these hematological parameters.

**Table 4.** Correlation analysis between summary index and biochemical and hematological parameters in older adults (n=70)^a^.

Parameters	Spearman ρ	*P* value
Biochemicals		
GOT^b^ (UI/L)	−0.08	.4647
Alkaline phosphatase (UI/L)	−0.09	.4103
LDH^c^ (UI/L)	0.01	.9138
Bilirubin (mg/dL)	0.33	.0049
Glycemia (mg/dL)	0.32	−.1196
Uric acid (mg/dL)	0.09	.4329
Total cholesterol (mg/dL)	−0.13	.2537
BUN^d^ (mg/dL)	−0.19	.0989
Calcium (mg/dL)	0.03	.7542
Phosphorus (mg/dL)	−0.10	.3741
Total proteins (mg/dL)	−0.11	.3265
Albumin (mg/dL)	0.10	.3683
CRP^e^ (mg/dL)	−0.08	.4932
Hematological		
ESR^f^ (mm/h)	−0.20	.0868
Serum iron (μg/dL)	0.27	.0272
TIBC^g^ (μg/dL)	−0.02	.8634
Transferrin saturation (%)	0.24	.0386
Hemoglobin (g/dL)	0.19	.1122
Hematocrit (%)	0.21	.0758
RBC^h^ count (x10^6^ cells/µL)	0.26	.0303
MCHC^i^ (%)	−0.07	.5511
MCV^j^ (fL)	−0.11	.3537
RDW^k^ (%)	0.23	.0462
WBC^l^ count (cells/µL)	−0.06	.5770
Platelet count (cells/µL)	0.07	.5245
MPV^m^ (fL)	0.07	.5626

a Nonparametric Spearman correlations were employed for all correlation analyses.

b GOT: glutamic-oxaloacetic transaminase.

c LDH: lactate dehydrogenase.

d BUN: blood urea nitrogen.

e CRP: C-reactive protein.

f ESR: erythrocyte sedimentation rate.

g TIBC: total iron-binding capacity.

h RBC: red blood cell.

i MCHC: mean corpuscular hemoglobin concentration.

j MCV: mean corpuscular volume.

k RDW: red blood cell distribution width.

l WBC: white blood cell.

m MPV: mean platelet volume.

### Nutritional Status Assessed by CONUT Score According to Frailty Status and Its Correlation With Urban Quality

**Figure 6.**
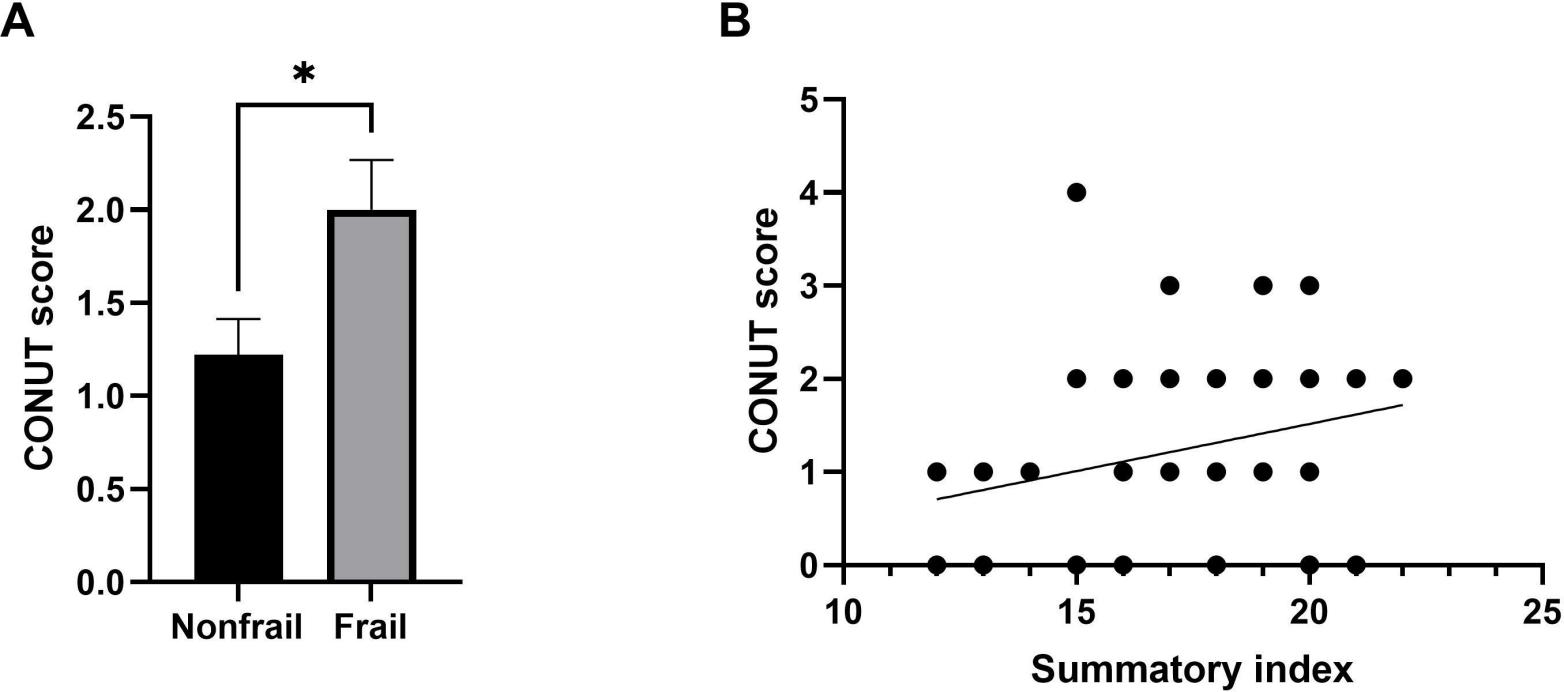
Relationship between nutritional status assessed using the CONUT score, frailty, and summary index in older adults. (A) Comparison of CONUT score based on frailty status according to the Frailty Trait Scale 5 Scale. (B) Linear regression analysis between CONUT score and summary index. Statistical analysis was performed by *t* test. *P<.05. CONUT: Controlling Nutritional Status.

Figure 6 shows a comparison of the CONUT score by frailty status and its correlation with urban quality, measured through the summary index. In Figure 6A, the frail group shows a significantly higher CONUT score (*P*=.0323), indicating poorer nutritional status. Meanwhile, Figure 6B displays the linear regression analysis between the CONUT score and the summary index, revealing a significant positive correlation between both variables (Spearman ρ=0.2513; *P*=.0359).

## Discussion

### Principal Findings

This study examined the association between urban quality and frailty in older adults by integrating geospatial analysis with nutritional and hematological or biochemical parameters. Key findings revealed that higher urban quality was significantly associated with altered hematological markers (eg, elevated bilirubin, serum iron, and RBC count), poorer nutritional status (as indicated by higher CONUT score), and increased frailty prevalence. Frail individuals living in areas of higher urban quality also exhibited lower handgrip strength, higher frailty scores, and greater proximity to emergency health centers, community centers, and exercise facilities. In summary, older adults residing in neighborhoods with the greatest proximity to services, parks, and health facilities were more frequently classified as frail and exhibited weaker muscle strength, poorer nutritional status, and modest increases in iron-related and erythrocyte markers, while inflammatory and renal-hepatic indicators remained largely unaffected. While these spatial patterns suggest potential links between urban environment and health outcomes in aging populations, the cross-sectional design limits causal inference. Because all exposures and outcomes were assessed at a single point in time, the cross-sectional design precludes establishing temporal precedence; therefore, observed associations cannot be interpreted as causal. Reverse causation is plausible, and residual confounding from unmeasured factors may persist despite statistical adjustment. Several potential sources of bias should also be acknowledged. First, recruiting participants through primary care clinics may overrepresent older adults who are relatively health-seeking, potentially underestimating the true prevalence of frailty (selection bias). Second, minor geocoding errors and reliance on single time-point biomarker measurements may lead to exposure and outcome misclassification. Given these limitations, we emphasize the need for prospective studies to further investigate these associations and clarify their causal pathways.

The results indicate that frailty prevalence in the studied cohort was 17.5% (44/251), which aligns with previous research reporting frailty rates ranging from 12% to 27% in Chilean older adults [19,22]. The higher BMI observed in frail individuals is consistent with studies suggesting that obesity exacerbates physical limitations and inflammation, contributing to frailty [23-25]. Although the frail group had a higher mean age, the difference was not statistically significant. Age is a well-known factor for frailty, with prevalence increasing in advanced age, particularly in individuals aged 80 years and older [26,27]. However, it is important to consider that environmental and lifestyle factors may play a more substantial role than age alone [3]. The lack of significant differences in frailty prevalence across spatial clusters suggests a uniform distribution of risk factors in the evaluated area. This also highlights the need to expand the geospatial analysis to different urban and rural areas to capture the variations in frailty prevalence and associated risk [8].

Consistent with the previous findings, no significant difference in frailty prevalence was observed across quartiles based on the summary index. However, Q4 did show a higher prevalence of frailty than Q1, although this difference did not reach statistical significance, suggesting that a larger sample size may be needed to confirm this trend. In addition, Q4 exhibited a higher FTS-5 score, indicating a greater level of frailty. These findings suggest that frail older adults may be more likely to reside in areas with better urban quality, potentially reflecting adaptive behaviors related to their health status and quality of life [28,29]. For example, improved access to natural environments (an important component of urban quality) has been associated with reduced health inequality linked to income deprivation, indicating that older adults may choose to reside in better quality urban areas to support their health [30]. Likewise, increased greenery in urban neighborhoods has been linked to better physical and mental health, which may encourage frail individuals to settle in or remain within such environments to mitigate health declines [31].

On the other hand, Q4 also showed a significant decrease in handgrip strength, which correlates with a high FTS-5 score and greater frailty prevalence. Reduced handgrip strength is a marker of poorer muscle condition and is associated with increased risk of sarcopenia, chronic diseases, and mortality [32]. These findings suggest that addressing sarcopenia could be a valuable strategy in high-accessibility areas when designing interventions to prevent frailty and promote healthy aging in older adults [33].

This study also found that frail individuals tended to live significantly closer to exercise facilities, community health centers, and emergency medical services, which may reflect adaptive behaviors in response to health care accessibility. While proximity to these facilities suggests that frail older adults may prioritize access to spaces supporting physical activity and social interaction, closeness to emergency and community health centers likely provides critical support for managing both acute and chronic health conditions [31,34]. It is important to note that in this study, exercise facilities were operationally defined as stadiums and green areas equipped with infrastructure for physical activity. This definition represents a limitation when interpreting the associations observed. Moreover, the significant correlation between the summary index and frailty scores reinforces the hypothesis of a close relationship between frailty and urban environment quality. While our findings indicate that frail individuals live nearer to health and recreational facilities, actual use of these resources may be constrained by unmeasured sociodemographic barriers. These may include gender-related mobility limitations (particularly in older women), migration status (eg, language barriers), disability, and digital literacy (eg, limited access to digital health services) [35-37]. Future geospatial studies should incorporate individual-level data on these factors to better assess accessibility gaps in urban aging populations.

### Limitations

In addition, some methodological limitations should be noted. The use of a summatory index, while straightforward and consistent with previous local studies [14], may oversimplify the relative importance of each facility type. More complex approaches (eg, weighted indices or principal component analysis) may offer additional insights in future research. Moreover, spatial autocorrelation and the modifiable areal unit problem were not formally addressed. These are inherent challenges in geospatial analyses and should be explored in future studies using alternative spatial units and subjective environmental assessments. It should also be noted that the spatial clusters used in this study were not constructed based on direct SES indicators. Rather, they were defined using historical, structural, and industrial characteristics of the region. Therefore, any interpretation of these clusters as SES proxies should be made with caution. This limitation highlights the need for future studies to integrate direct SES variables when analyzing spatial health inequalities.

The results support the notion that environmental factors significantly influence the health status of older adults, revealing modest but statistically significant correlation between frailty, urban quality, and select biochemical and hematological parameters. Given the study’s cross-sectional design, we emphasize the need for longitudinal research to confirm these associations and further explore causal mechanisms. The observed correlations offer valuable insights into the impact of urban environments on the nutritional status and metabolic health of older populations. Higher urban quality was associated with elevated bilirubin levels, which may reflect better liver function and more effective oxidative stress regulation. However, this correlation is modest and should be interpreted cautiously. Bilirubin, a well-established antioxidant, has been linked to reduced cardiovascular risk and lower systemic inflammation in prior studies [38]. Urban environments with better infrastructure are often associated with healthier lifestyles, including increased physical activity and dietary habits that support liver health [17]. Moreover, the positive correlation between urban quality and serum iron, RBC count, transferrin saturation, and RDW indicates a tentative association with certain hematological parameters [39]. These indicators are essential for evaluating anemia and overall oxygen transport capacity, both of which are critical determinants of functional status and frailty in older adults [18]. Conversely, poor urban quality, characterized by limited access to nutrient-dense food and inadequate health care services, has been linked to higher risks of anemia and micronutrient deficiencies [40]. Our findings are consistent with previous studies suggesting that the built environment influences dietary patterns, which may in turn affect hematological and biochemical health markers [16].

Our results also reveal an association between frailty and higher CONUT scores, underscoring the close interplay between nutritional status and frailty. Malnutrition is a well-established contributor to frailty, and the CONUT score has been validated as a reliable marker of nutritional risk in frail older adults [21,41]. Our findings indicate that frail individuals tend to have poorer nutritional status, which aligns with previous research linking malnutrition to sarcopenia, immune dysfunction, and increased morbidity [19,42,43]. The observed association between elevated CONUT scores and frailty also supports evidence connecting malnutrition to chronic inflammation and sarcopenia, key biological pathways underlying frailty [21,41]. While our study included C-reactive protein as an inflammatory marker (showing no significant correlation with urban quality), future studies should incorporate proinflammatory cytokines such as interleukin-6 and tumor necrosis factor α to more comprehensively assess inflammatory dysregulation [41]. It is important to note that the CONUT score’s reliance on serum albumin (a negative acute-phase reactant) may reflect both malnutrition status and subclinical inflammation, complicating its interpretation. The cutoff values used (albumin <3.5 g/dL, lymphocytes <1500/mm³, and total cholesterol <160 mg/dL) are based on the original CONUT validation [21]; however, their applicability and sensitivity to aging populations warrant further investigation. Interestingly, this positive correlation observed between urban quality and CONUT scores may indicate that better urban infrastructure supports improved nutritional status. Access to fresh food markets and community resources likely promotes better dietary intake, helping to reduce malnutrition and the burden of nutrition-related chronic diseases [44,45]. In contrast, lower urban quality may be associated with food insecurity and limited access to nutrient-dense foods, increasing the risk of frailty through inadequate protein and micronutrient intake [40,46].

It is important to emphasize that these findings highlight the need to address sociourban inequities in age-friendly urban planning. As demonstrated, targeted interventions, such as increasing opportunities for physical activity in low SES areas, can help mitigate health disparities. This suggests that improvements in urban quality should prioritize vulnerable subgroups to reduce frailty gradients across populations [47].

Notably, the paradoxical finding that frailer individuals resided closer to fresh food markets and yet exhibited poorer nutritional status (as indicated by higher CONUT scores) warrants further investigation. Although proximity to food sources is often assumed to enhance dietary intake, various barriers (such as mobility limitations, financial hardships, or lack of social support) may prevent frail older adults from effectively using these resources. This observation is consistent with prior studies indicating that physical access alone does not ensure food security or healthy eating behaviors, particularly among socially and physically vulnerable populations [40,45]. Similarly, the observed proximity of frail individuals to exercise facilities may reflect adaptive strategies to manage functional decline; however, actual engagement in physical activity may be constrained by comorbidities, pain, or reduced mobility [33]. These findings underscore the importance of complementing urban infrastructure with targeted, supportive interventions that bridge the gap between accessibility and actual utilization among frail older adults.

### Conclusions

Our research demonstrates that among older adults in Chile, urban quality has a significant impact on their metabolic health, nutritional status, and frailty. Frail individuals residing in areas with higher urban quality exhibited lower handgrip strength and higher frailty scores. In addition, they tended to live closer to emergency rooms, community health centers, and exercise facilities. While this spatial pattern suggests potential accessibility advantages, its relationship with frailty requires further investigation. Moreover, higher CONUT scores in frail individuals and positive correlations between urban quality and key biochemical or hematological parameters indicate that better urban environments may support healthier metabolic and nutritional profiles. Given the cross-sectional design and localized scope of this study, future research should incorporate longitudinal approaches and larger, more diverse cohorts to confirm these associations and explore the underlying mechanisms. Finally, urban planning strategies promoting age-friendly environments may help mitigate frailty risk; however, longitudinal studies are needed to clarify the roles of selective migration, aging in place, and disparities in urban development in shaping these outcomes.

## Abbreviations

CONUT: Controlling Nutritional Status
FTS: Frailty Trait Scale
FTS-5: Frailty Trait Scale 5
GIS: Geographic Information System
RBC: red blood cell
RDW: red blood cell distribution width
SES: socioeconomic status
